# 697. Outcomes of Tigecycline Use for *Clostridioides difficile* Infection: A Case Series of 28 Patients

**DOI:** 10.1093/ofid/ofab466.894

**Published:** 2021-12-04

**Authors:** Emma C Phillips, Cirle A Warren, Gregory Madden

**Affiliations:** 1 University of Virginia School of Medicine, Charlottesville, VA; 2 University of Virginia, Charlottesville, VA; 3 Division of Infectious Diseases & International Health, Charlottesville, VA

## Abstract

**Background:**

*Clostridioides difficile* infection remains a highly morbid or lethal condition in an unacceptably large proportion of patients. To date, there are limited and conflicting data to support the use of tigecycline for *C. difficile* infection and the optimal stratification approach, timing (i.e., initial vs. salvage therapy), and duration are unclear.

**Methods:**

We describe in detail a retrospective cohort of 28 *C. difficile* inpatients treated with tigecycline at UVA Medical Center. We stratify each patient by the Infectious Diseases Society of America’s guidelines on severity of infection and detail the timing and duration of tigecycline therapy in each case. We further characterize the effect of tigecycline on 90-day mortality and recurrence.

**Results:**

9/28 (32.1%) patients were treated with tigecycline for fulminant (presence of hypotension, shock, ileus, or megacolon), and 12/28 (42.9%) for severe (white blood cell count over 15x10^9^/L or creatinine over 1.5mg/dL) *C. difficile* infection. Tigecycline was used in all cases in combination with oral vancomycin +/- metronidazole. The average duration of therapy was 7.6 days, with tigecycline as initial therapy (use within the first 72 hours of the start of directed antimicrobial therapy) in 7/28 (25%) cases. 90-day mortality occurred in 10/26 (35.7%) patients (two did not reach 90-day follow-up), all 10 of which were in-hospital mortalities and 5/10 (50%) occurred in patients with fulminant infection. 7 of the 16 (43.8%) surviving patients that reached 90-day follow-up had recurrent *C. difficile* infection.

**Conclusion:**

Patients selected for treatment with tigecycline for *C. difficile* infection suffered a high rate of in-hospital mortality, especially among the significant proportion with fulminant disease. The rate of recurrent infection was substantial, contrary to some reports of reduced recurrence with tigecycline from the literature. The outcomes of tigecycline (as adjunct or monotherapy) for treatment of severe/fulminant and refractory infection versus standard treatments warrant further retrospective analysis and the benefit of tigecycline in these settings remains to be proven in well-controlled clinical trials.

**Disclosures:**

**All Authors**: No reported disclosures

